# Side-effects of domestication: cultivated legume seeds contain similar tocopherols and fatty acids but less carotenoids than their wild counterparts

**DOI:** 10.1186/s12870-014-0385-1

**Published:** 2014-12-20

**Authors:** Beatriz Fernández-Marín, Rubén Milla, Nieves Martín-Robles, Erwann Arc, Ilse Kranner, José María Becerril, José Ignacio García-Plazaola

**Affiliations:** Department of Plant Biology and Ecology, University of the Basque Country (UPV/EHU), Apdo. 644, 48080 Bilbao, Spain; Departamento de Biología y Geología, Área de Biodiversidad y Conservación, Escuela Superior de Ciencias Experimentales y Tecnología, Universidad Rey Juan Carlos, c/Tulipán s/n, Móstoles, 28933 Spain; Institute of Botany, and Center for Molecular Biosciences Innsbruck, University of Innsbruck, Sternwartestraße 15, A-6020 Innsbruck, Austria

**Keywords:** Carotenoid, Domestication, Fatty acid, Seed, Tocopherol, Wild relative

## Abstract

**Background:**

Lipophilic antioxidants play dual key roles in edible seeds (i) as preservatives of cell integrity and seed viability by preventing the oxidation of fats, and (ii) as essential nutrients for human and animal life stock. It has been well documented that plant domestication and post-domestication evolution frequently resulted in increased seed size and palatability, and reduced seed dormancy. Nevertheless, and surprisingly, it is poorly understood how agricultural selection and cultivation affected the physiological fitness and the nutritional quality of seeds. Fabaceae have the greatest number of crop species of all plant families, and most of them are cultivated for their highly nutritious edible seeds. Here, we evaluate whether evolution of plants under cultivation has altered the integrated system formed by membranes (fatty acids) and lipophilic antioxidants (carotenoids and tocopherols), in the ten most economically important grain legumes and their closest wild relatives, i.e.: *Arachis *(peanut), *Cicer *(chickpea), *Glycine *(soybean), *Lathyrus*(vetch), *Lens *(lentil), *Lupinus *(lupin), *Phaseolus *(bean), *Pisum *(pea), *Vicia *(faba bean) and *Vigna *(cowpea).

**Results:**

Unexpectedly, we found that following domestication, the contents of carotenoids, including lutein and zeaxanthin, decreased in all ten species (total carotenoid content decreased 48% in average). Furthermore, the composition of carotenoids changed, whereby some carotenoids were lost in most of the crops. An undirected change in the contents of tocopherols and fatty acids was found, with contents increasing in some species and decreasing in others, independently of the changes in carotenoids. In some species, polyunsaturated fatty acids (linolenic acid especially), α-tocopherol and γ-tocopherol decreased following domestication.

**Conclusions:**

The changes in carotenoids, tocopherols and fatty acids are likely side-effects of the selection for other desired traits such as the loss of seed dormancy and dispersal mechanisms, and selection for seed storability and taste. This work may serve as baseline to broaden our knowledge on the integrated changes on crop fitness and nutritional quality following domestication.

**Electronic supplementary material:**

The online version of this article (doi:10.1186/s12870-014-0385-1) contains supplementary material, which is available to authorized users.

## Background

Plant domestication by humans likely started in the Middle East approximately 13,000 years ago, although geographically and temporarily separated events occurred at an estimated 13 locations around the World 13,000 to 3,000 years before present [1]. Both, initial domestication *sensu stricto* and post-domestication evolution have influenced morphological and physiological traits of crops [2]. Most probably, taste, yield, storability, and heritability of desired traits were major determinants in the decision making of early farmers, similarly to present day preferences [2]. In addition, for some crops, unconscious selection of physical characters [3] or invisible nutritional traits, such as high free tryptophan content that determines brain serotonin synthesis, could have been involved in the decision of prehistoric humans to select species for farming [4]. Genetic changes upon domestication and crop evolution related to the profitability of farming operations are well documented, such as diminution of seed dispersal mechanisms and seed dormancy, increases in seed size and other plant traits bearing on agronomic performance [5-9].

In contrast, less attention has been paid to the effects of domestication on the nutritional value of seeds. The scarce literature available has focused on protein and amino acid contents [4,10-13] and very little information exists on other important nutritional traits such as starch [14] or oils [15]. Carotenoids, tocopherols and fatty acids (FAs) play crucial roles in edible seeds (*i*) as essential dietary elements comprising vitamin precursors, antioxidants and essential ω-3 FAs, required for human and livestock nutrition, and (*ii*) for the seed they are required to support membrane integrity, contributing to seed fitness. Despite their pivotal roles for seed viability and nutrition, it is unknown if these metabolites were selected for by the early farmers, and if they were changed upon post-domestication evolution.

Upon seed storage, seed viability can be compromised if the antioxidant system fails, leading to the accumulation of reactive oxygen species (ROS). Evidence is emerging that ROS contribute to oxidative signalling required for plant growth and development as well as cell death [16-18]. The accumulation of ROS can result in seed death when it occurs in critical amount or location such as in the embryo [18-20]. Moreover, ROS can rapidly react with other molecules, including FAs, resulting in lipid peroxidation, phospholipid degradation and loss of membrane integrity [19,21,22]. The composition of membrane FAs has been correlated with seed storability [23]. Polyunsaturated fatty acids (PUFAs) are particularly sensitive to lipid peroxidation [24]. Lipophilic antioxidants protect cells from lipid peroxidation and hence, from membrane damage, and high tocopherol contents have been positively correlated with seed viability [25-27]. The contribution of carotenoids to the antioxidant activity of seeds is poorly understood compared to their roles in photosynthetic tissues, where they are accessory photosynthetic pigments with antioxidant capacity involved in thermal dissipation of excess light energy and ROS scavenging [28]. In seeds, carotenoids are precursors of the phytohormone abscisic acid (ABA), which is a positive regulator of seed dormancy [29,30]. Moreover, as colourful molecules, they increase attractiveness to seed dispersers [31].

In humans, tocopherols and carotenoids have roles as antioxidants, as they do in plants, but with extended impacts on human physiology. Carotenoids prevent oxidation in the macula of the eye, and deficiency in carotenoids can cause blindness [32,33]. Tocopherols play crucial roles in anti-inflammatory processes [34,35] and deficiency results in a range of disorders, including neuromuscular problems [36] and coronary diseases [37]. Generally, a high intake of unsaturated fatty acids (UFAs) relative to saturated fatty acids (SFAs) prevents coronary heart disease [38].

Considering the highly beneficial effects of carotenoids, tocopherols and UFAs on human health, and based on the trends already found for other nutritionally relevant traits [4], one would intuitively expect that our extant crops contain higher contents than their wild progenitors. However, FAs and tocopherols are invisible to the human eye, in contrast to the colourful carotenoids. Seed colour is an agronomic trait subject to directional selection [9]. Hence, a selective change in the coloured carotenoids could have occurred independently of the changes in the invisible tocopherols and FAs.

We chose a set of the ten most important species for human and livestock feeding in the Fabaceae family and their closest wild relatives to study the modification of carotenoids, tocopherols and FAs upon domestication. This family was chosen (*i*) because of its agricultural and economic importance: with 41 domesticated species, the legumes have the greatest number of crop species of all plant families, most of which are cultivated for their highly nutritious edible seeds [6,7]; (*ii*) because legume seeds appear to contain higher levels of carotenoids than the edible seeds of other plant families [39]. Our work is intended to provide baseline information on the impact of domestication and post-domestication evolution on carotenoids, tocopherols and FAs, which could be key contributors to future improvements of the nutritional value and the storability of edible seed crops.

## Methods

### Plant material

Ten of the most widely consumed grain legume species and their ten corresponding most closely related wild relatives were selected for this study (Table 1). The ten selected species account for 97% of total harvest tons and 94% of World cultivated Ha for legume production (FAO 2013, http://faostat.fao.org). For detailed information, literature regarding wild progenitor assignment and for suppliers see Additional file 1. All seed lots were submitted to the same protocol before analyses. Seeds were transported from the providers (where they were stored at low temperature and relative humidity) in sealed envelopes at low humidity. Once in the lab, seed material was kept over silica gel at −20°C until use. Seeds were then ground to a fine powder with mortar and pestle using liquid nitrogen before extraction. Five independent biological replicates (2 to 10 seeds per replicate, depending on seed size, to obtain at least 75 mg of dry material) were produced for each seed lot. Each replicate was analysed for carotenoids, tocochromanols and FAs contents (except for *Cicer arietinum* and *Lens culinaris*, for which a separate set of seeds was used for FA analysis).

**Table 1:** **Grain legumes used in this study and their closest wild relatives (D, domesticated; W, wild)**

**Common name**	**Domestication status**	**Scientific name**	**Country of origin**
Peanut	W	*Arachis monticola* Krapov. & Rigoni	Argentina
	D	*Arachis hypogaea* L.	China
Chickpea	W	*Cicer reticulatum* Ladiz.	Turkey
	D	*Cicer arietinum* L.	Spain
Soybean	W	*Glycine soja* Sieber & Zucc.	Russia
	D	*Glycine max* (L.) Merrill	Japan
Vetch	W	*Lathyrus cicera* L.	Spain
	D	*Lathyrus sativus* L.	Greece
Lentil	W	*Lens culinaris* subsp*. orientalis* (Boiss.) Ponert	Cyprus
	D	*Lens culinaris* Medik.	Spain
Lupin	W	*Lupinus luteus* L.	Spain
	D	*Lupinus luteus* L.	Spain
Bean	W	*Phaseolus lunatus* L.	Peru
	D	*Phaseolus lunatus* L.	Colombia
Pea	W	*Pisum sativum* L.	Greece
	D	*Pisum sativum* L.	Spain
Faba bean	W	*Vicia narbonensis* L.	Spain
	D	*Vicia faba* L.	Spain
Cowpea	W	*Vigna unguiculata* subsp*. unguiculata* (L.) Walp	Nigeria
	D	*Vigna unguiculata* (L.) Walp.	USA

Country of origin refers to the country where the seeds were collected.

### Carotenoids analysis

Approximately 20 mg dry mass (DM) (exact mass recorded) of seed powder per replicate were used for carotenoids analyses. Carotenoids were extracted in 250 μL of 95% acetone containing 0.1 g L^−1^ of CaCO_3_ at ≤ 4°C using cold racks (IsoPack, Eppendorf 022510258 IsoTherm®, USA) and centrifuged for 10 min at 16,100 g. The supernatant was collected and the pellet resuspended in 250 μL of 100% acetone and centrifuged for 10 min at 16,100 g. The two supernatants were combined and filtered through a 0.2 μm PTFE filter (Teknokroma, Barcelona, Spain). Carotenoids were separated by HPLC (Waters, Barcelona, Spain) on a reversed-phase C18 column (Waters Spherisorb ODS1, 4.6 x 250 mm, 5 μm particle size, Milford, Massachusetts, USA) and detected with a Photodiode Array Detector (Waters 996, Waters, Barcelona, Spain), following the method of García-Plazaola and Becerril [40,41].

### Tocochromanols analysis

Approximately 20 mg DM per replicate were used for the analyses of tocochromanols (i.e., tocopherols and tocotrienols). Tocochromanols were extracted at ≤ 4°C (as above) in 500 μL of 99% heptane and centrifuged for 10 min at 16,100 g. The supernatant was collected, the pellet resuspended in 500 μL of 99% heptane and centrifuged for 10 min at 16,100 g. The two supernatants were combined and filtered through a 0.2 μm PTFE filter (Teknokroma, Barcelona, Spain). Tocochromanols were separated by HPLC on a Diol column (Supelcosil LC-Diol, 4.6 × 250 mm, 5 μm particle size, Supelco Analytical, Sigma-Aldrich, Bellefonte, USA) using a modified method of Bagci et al. [42]. The system was operated with heptane:tert.-butylmethyl ether 97.5:2.5 v:v as an eluant at a flux of 1 mL min^−1^, and tocochromanols were detected with a Scanning Fluorescence Detector (Waters 474, Waters, Barcelona, Spain) set to an excitation wavelength of 295 nm and an emission wavelength of 330 nm. Tocochromanols were quantified using calibration curves of α-, β-, γ-, and δ-tocopherol and α-, β-, γ-, and δ-tocotrienol standards (Calbiochem, Darmstadt, Germany).

### Total fatty acids analysis

Fatty acids were derivatised to fatty acid methyl esters (FAMEs) as described by Li-Beisson et al. [43]. Briefly, around 20 mg of freeze-dried seed powder were treated with 2.64 mL of methanol:toluene:sulfuric acid 10:3:0.25 v:v:v containing 0.01% (w:v) butylated hydroxytoluene. 200 μg of heptadecanoic acid (C17:0, dissolved in hexane) were added simultaneously as internal standard. Samples were incubated at 80°C for 90 min with constant agitation before adding 1 mL of hexane and 3 mL of 0.9% NaCl (w:v). Samples were vigorously mixed before centrifugation for 10 min at 3,000 g. The supernatant was transferred to autosampler vials and kept at −20°C if not analysed immediately. FAMEs were separated using a Trace 1300 gas chromatograph (GC) (Thermo-Scientific, USA) on a 30 m FAMEWAX column (Restek #12497, Bellefonte, USA) and detected using a TSQ 8000 triple quadrupole detector (Thermo-Scientific, Waltham, USA) operated in full scan mode (50 – 550 m/z). The temperature gradient was first set to 120°C for 1 min, then increased by 9°C per min up to 200°C, and finally increased by 3°C per min up to 230°C, which was held for 6 min with a carrier gas flow of 1 mL min^−1^ of helium. The ion source temperature was set to 250°C and the transfer line to 240°C. The split ratio was adjusted from 20 to 150 depending on total fatty acid abundance. A commercial FAME mix (Sigma Aldrich ref. 18919, Missouri, USA) was used to confirm the identities of the FAs. External standards of palmitic, stearic, oleic, linoleic and linolenic acid were used in conjunction with the internal standard to estimate the total amount of each fatty acid. Data analysis was performed using the Xcalibur software (Thermo-Scientific, Waltham, USA).

### Data processing and statistics

Variations (Δ%) in the contents of carotenoids, tocopherols and FAs following domestication were calculated as percentages of the contents in the wild species as follows [(domesticated-wild)/wild*100]. Two-way analysis of variance (ANOVA) was used to evaluate the effects of the genus and of the domestication status (domesticated or wild) and their random slope on total contents of carotenoid, tocopherol, oil, SFAs, MUFAs and PUFAs. The genus was used as a random-effect factor, and the domestication status was a fixed-effect factor. One-way ANOVA with Dunnett C test as *pot hoc* was used to check for differences in total carotenoids and total tocopherol contents, in FAs, total seed oil content and in the contents of SFAs, MUFAs and PUFAs between each grain legume and its corresponding wild relative. Pearsons’ correlation was used to analyse the relationship between the variation of lipophilic seed antioxidants and FAs contents in grain legumes and their wild relatives. Statistical analyses were performed using the SPSS 19.0 statistical package.

## Results

### Lipophilic antioxidants

**Figure 1:**
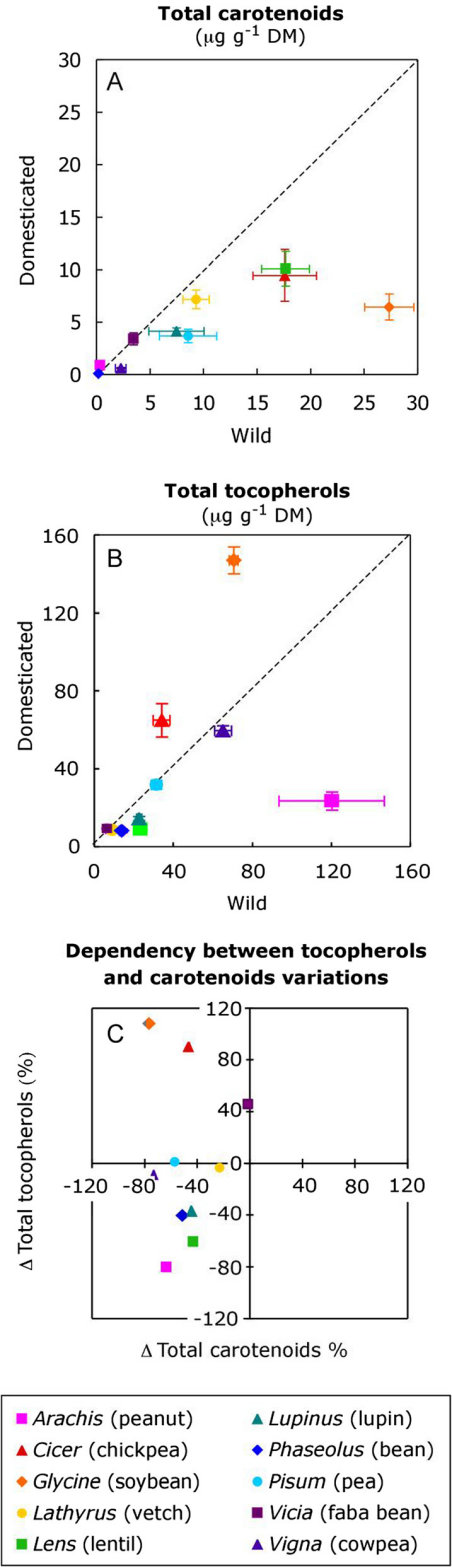
**Effects of domestication on total seed carotenoids and tocopherols contents. (A)** Bisector plots representing total carotenoids contents and **(B)** total tocopherols contents of grain legumes plotted against their closest wild relatives. Data points below the dotted line (y = x) indicate that the domesticated species contained less carotenoids or tocopherols than their wild relatives. Data points show arithmetic means ± SE (n = 5). Note that the contents of total carotenoids significantly decreased following domestication (panel A; F_domestication_ = 49.0, *P* < 0.001 and F_random solpe_ = 32.3, *P* < 0.001, two-way ANOVA) but no general pattern was found for tocopherols (F_domestication_ = 0.039, *P* = 0.843). **(C)** Dependency between the variations in total seed carotenoids and tocopherols contents following domestication. Variations (Δ%) are expressed as percentages of the contents in the wild species. Data points represent means (n = 5). Pearson’s correlation was not significant (*P* = 0.954), indicating that the changes in carotenoids and tocopherols occurred independently.

Overall, the total seed carotenoids contents of domesticated plants was significantly lower than that of their wild counterparts (two-way ANOVA; *P* < 0.001, Figure 1A). Total carotenoids contents was lower in the domesticated plants of the ten genera analysed (Table 2) whereby the extent of the difference with their wild progenitors was genus-dependent (P < 0.001, Figure 1A). Pairwise comparison showed that this difference was significant in six of the ten genera analysed (one-way ANOVA; Table 2) and greatest in *Glycine* (Figure 1A). In contrast, total tocopherols did not appear to show consistent differences between wild and domesticated plants (Figure 1B and Table 2). Tocopherols contents were significantly (*P* < 0.05) lower in some of the grain legumes (*Arachis, Lens and Lupinus*) whereas in other cases they were lower in the wild relatives (*Glycine, Cicer, Vicia*) (Figure 1B). Accordingly, no dependency was found between the changes in total tocopherols and total carotenoids (Figure 1C).

**Table 2:** **Concentration of total carotenoids and total tocopherols on domesticated (D) and wild (W) legume seeds**

	**Total carotenoids (μg g** ^**−1**^ **DM)**			**Total tocopherols (μg g** ^**−1**^ **DM)**		
**Crop**	**W**	**D**	**% Δ**	**W**	**D**	**% Δ**
*Arachis*	0.9 ± 0.2	0.3 ± 0.4	−63.3	120.2 ± 26.7	23.5 ± 4.6	−80.5*
*Cicer*	17.6 ± 3.0	9.4 ± 2.5	−46.3*	34.1 ± 4.2	64.9 ± 8.5	90.3*
*Glycine*	27.3 ± 2.3	6.4 ± 1.2	−76.4*	70.6 ± 2.3	146.9 ± 6.9	108.2*
*Lathyrus*	9.3 ± 1.2	7.2 ± 0.9	−23.0	9.1 ± 0.5	8.7 ± 0.9	−3.6
*Lens*	17.7 ± 2.2	10.1 ± 1.7	−42.9*	23.3 ± 2.0	9.1 ± 0.6	−60.7
*Lupinus*	7.5 ± 2.6	4.1 ± 0.3	−44.7*	22.7 ± 1.3	14.5 ± 1.0	−36.3*
*Phaseolus*	0.2 ± 0.0	0.1 ± 0.1	−51.5	13.8 ± 1.0	8.2 ± 0.9	−40.1*
*Pisum*	8.6 ± 2.7	3.7 ± 0.7	−57.0*	31.5 ± 2.3	31.8 ± 2.1	1.1
*Vicia*	3.5 ± 0.4	3.4 ± 0.6	−1.8	6.4 ± 0.6	9.4 ± 0.2	45.7*
*Vigna*	2.3 ± 0.5	0.6 ± 0.1	−73.2*	65.2 ± 4.3	59.5 ± 2.6	−8.8

The percentage of variation during domestication is also shown [% Δ = (D-W)/W^.^100] for both carotenoids and tocopherols. Values are means ± SE (n = 5). Asterisks denote significant differences between grain legumes and their wild relatives (One-way ANOVA, *P* < 0.05).

**Figure 2:**
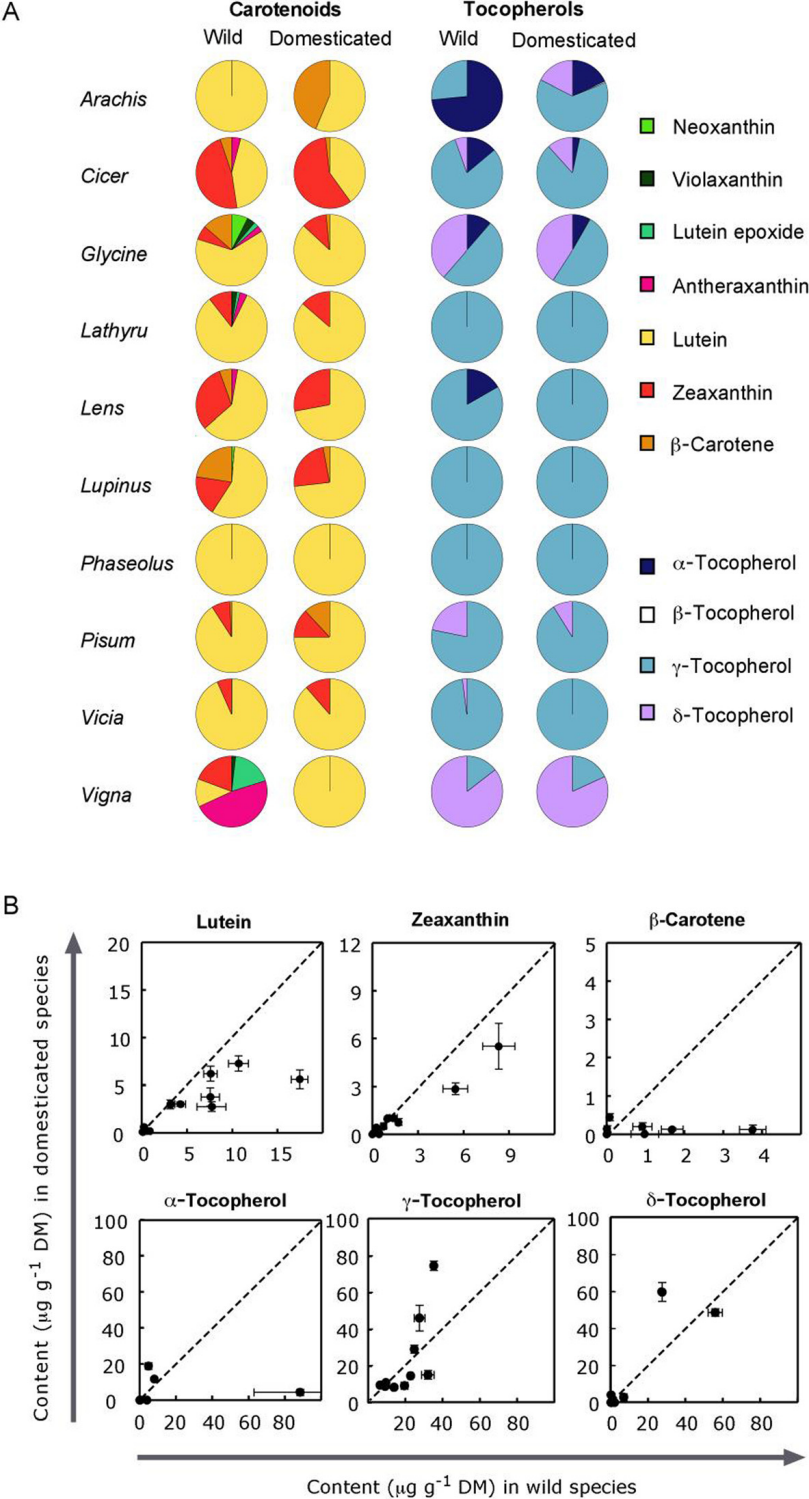
**Composition of carotenoids and tocopherols in seeds of grain legumes and their closest wild relatives. (A)** Individual carotenoids and tocopherols are expressed as proportions of total contents. Data are means (n = 5). **(B)** Bisector plots representing the absolute contents of carotenoids and tocopherols in grain legumes plotted against their closest wild relatives (only compounds shared among most species are shown). Data points below the dotted line (y = x) indicate that grain legumes contained less seed carotenoids or tocopherols than their wild relatives.

The composition of individual carotenoids and tocopherols in grain legumes also differed from that of their wild relatives (Figure 2). Lutein was the predominant carotenoid in all the species, followed by zeaxanthin and β-carotene (Figure 2A), except for the *Vigna* wild relative. Of all tocopherols, γ-tocopherol was the most abundant isoform in all species, except for *Vigna* and *Arachis monticola*, where δ − tocopherol and α-tocopherol were the main isoforms, respectively. No tocotrienols were detected in any seed lot. As a proportion of total seed carotenoids, lutein was higher in the grain legumes than in their wild relatives, except for *Arachis.* When present, lutein epoxide, neoxanthin, violaxanthin, and antheraxanthin as a proportion of total carotenoids were lower in domesticated legume seeds (Figure 2A). As a proportion of total tocopherols, α-tocopherol was lower whereas γ-tocopherol was higher in domesticated plants (Figure 2A). The absolute content of lutein was lower in grain legumes than in their wild relatives, accounting for the lower amount of total carotenoids, whereas no consistent difference between grain legumes and their wild counterparts was found for the individual tocopherols (Figure 2B, Additional file 2, Additional file 3).

### Fatty acids

**Figure 3:**
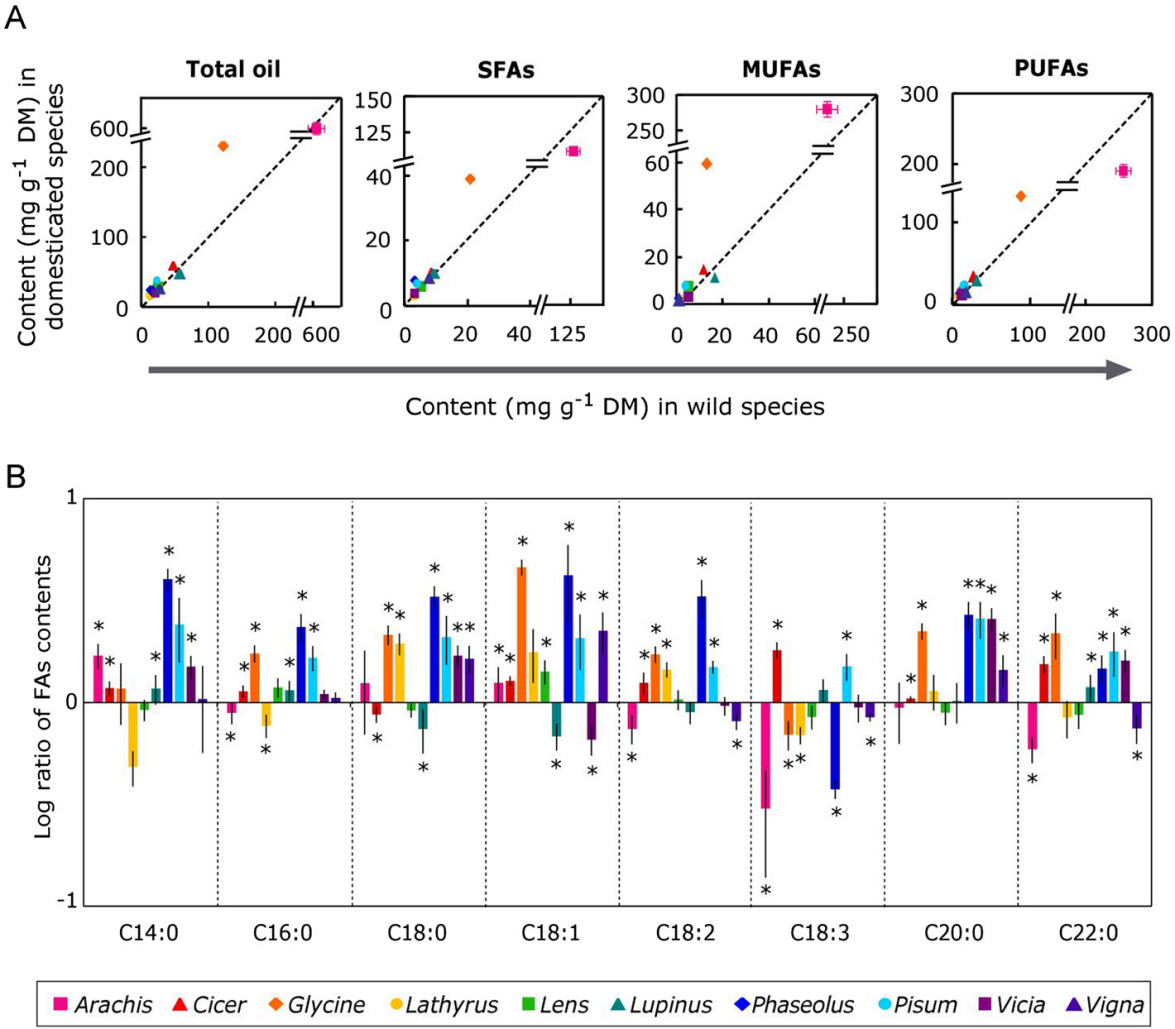
**Content and composition of fatty acids in the seeds of grain legumes and their closest wild relatives. (A)** Bisector plots representing the absolute contents of total oils, saturated fatty acids (SFAs), monounsaturated fatty acids (MUFAs) and polyunsaturated fatty acids (PUFAs) in grain legumes plotted against the contents in their closest wild relatives. Data points above the dotted line (y = x) indicate that grain legumes contained higher contents than their wild relatives. Data points show means ± SE (n ≥ 4) (the small standard errors are not appreciable in the figure for most of the species). No general pattern of change was found for FA following domestication (TotalOil F_domestication_ = 1.274, *P* = 0.288; SFAs F_domestication_ = 0.199, *P* = 0.666; MUFAs F_domestication_ = 2.43, *P* = 0.153; PUFAs F_domestication_ = 0.011, *P* = 0.920; two-way ANOVA). **(B)** Relative abundances of the main seed fatty acids (FAs) in grain legumes compared to their closest wild relatives (log_10_ ratios), showing which fatty acids increased (bars above zero) and which decreased (bars below zero) following domestication. FAs shown are: myristic acid (C14:0), palmitic acid (C16:0), stearic acid (C18:0), oleic acid (C18:1), linoleic acid (C18:2), linolenic acid (C18:3), arachidic acid (C20:0) and behenic acid (C22:0). Asterisks indicate significant differences between grain legumes and their wild relatives (one-way ANOVA, *P* < 0.05).

**Figure 4:**
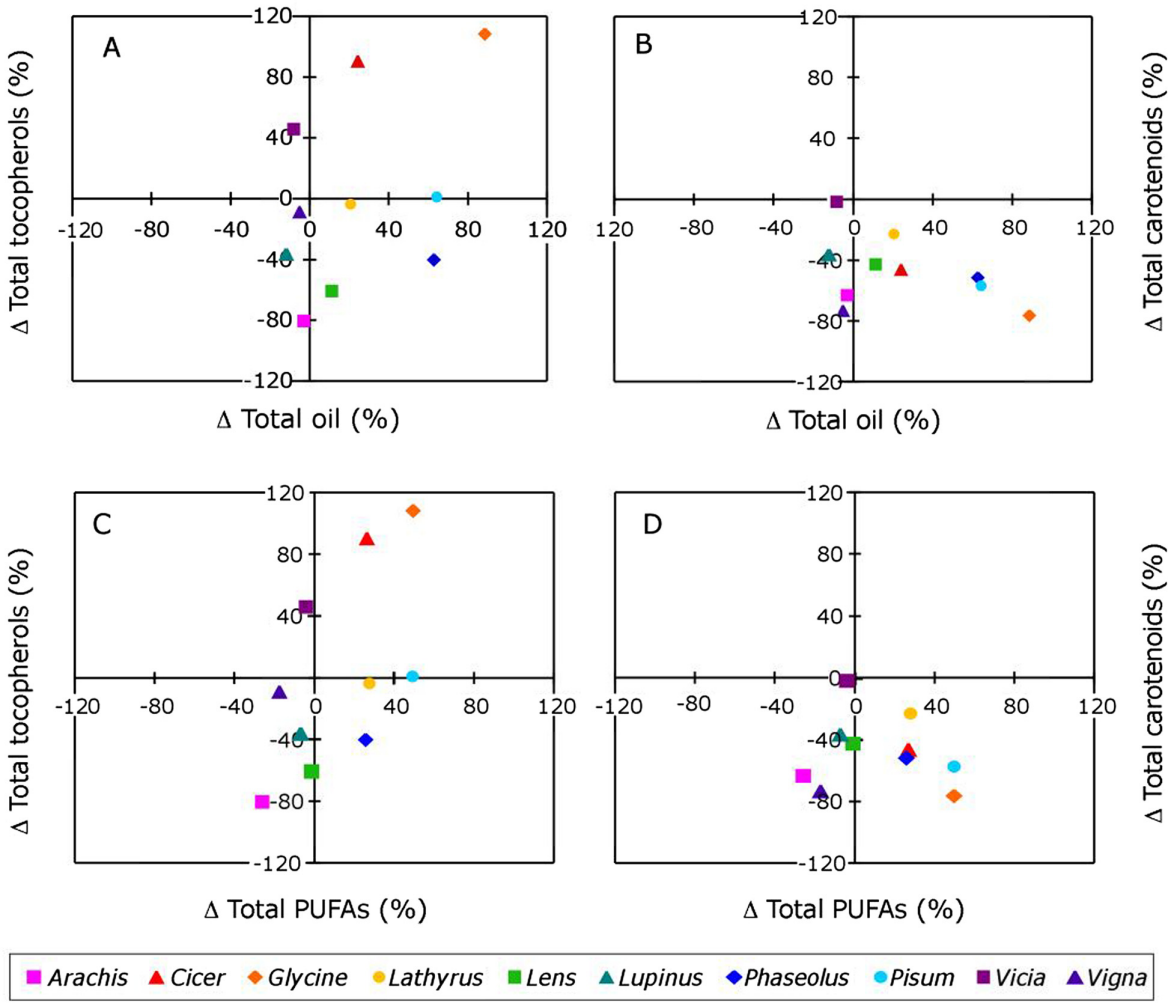
**Dependency between the variations in seed fatty acids and carotenoids and tocopherols following domestication.** Variations (Δ%) are expressed as percentages of the contents in the wild species. In **(A)** and **(B)** the variations in total oil content are plotted against variations in total tocopherols and carotenoids, respectively. In **(C)** and **(D)** the variations in total PUFAs are plotted against the variations in total tocopherols and carotenoids, respectively. Data are means (n ≥ 4). No significant correlation was found whereby the best correlation was observed between total tocopherols and PUFAs (Pearson’s correlation coefficient: 0.59; *P* = 0.07).

As found for tocopherols, total seed oil content did not appear to differ consistently between grain legumes and their wild relatives (Figure 3A, Additional file 4). Total oil content tended to be higher (by up to 1.65 fold) in domesticated *Cicer*, *Glycine, Lathyrus, Phaseolus,* and *Pisum,* slightly lower in domesticated *Lupinus* and showed no significant difference in the other four genera (Additional file 4). Most FAs were higher in domesticated grain legumes except linolenic acid, which was lower in the domesticated plants of all genera but *Cicer* and *Pisum* (Figure 3B). Fatty acids composition also did not show consistent differences among the analysed species between wild and domesticated plants (Figure 3, Additional file 4) and no dependence was found between the variations in FAs and in seed carotenoids and tocopherols contents following domestication (Figure 4). Although no significant, the best relationship was observed between total tocopherols and PUFAs (Pearson’s correlation coefficient: 0.59; P = 0.07, Figure 4C).

## Discussion

The advent of agriculture led to the transition of human societies from hunter-gatherers to early farmers, but it is not fully understood how domestication impacted upon the nutritional traits of edible seeds. By comparing crops with their close wild relatives, recent studies have provided new insights into biometric and physiological changes on the seed and the whole plant level [44,45]. We have used this approach to study the ten economically most important legume species cultivated for their edible seeds, accounting for 24.4% of Fabaceae domesticated species (10 out of the 41 domesticated species within the Fabaceae family [7]). These ten species were domesticated in several independent domestication centres [1] and are now cultivated on 190 million hectares of land globally (http://faostat.fao.org). Domestication implied a complex evolution process and although we do not address here the influence of environment on seed properties, our data suggest that domestication led to unexpected changes in carotenoids, tocopherols and FAs that are discussed with a view to seed quality and future breeding opportunities.

### Carotenoids, seed dormancy and dispersal

Although one might intuitively expect that compounds that are beneficial to human health are more concentrated in crops than in their wild relatives, total seed carotenoids decreased following domestication in all ten genera studied (Figure 1, Table 2). Two possible explanations for this unexpected finding are related to crop management requirements. Firstly, genotypes defective in autodispersal mechanisms were frequently selected for seed crops [1,2,9], i.e.: in many grain legumes, domestication involved the selection of genotypes lacking pod shattering mechanisms, with subsequent dependence on humans for dissemination [46]. Among others, endo- and exo-zoochory are important mechanisms that legumes use for seed dispersal [47,48]. Carotenoid derivatives are precursors of volatile scent and aroma constituents, such as geranyl acetone and β-ionone, which attract animals [49,50] and carotenoids may also signal maturity to potential seed predators and dispersers [51]. Following this line of reasoning, seeds with lower carotenoid contents would have been less attractive to seed dispersers.

Secondly, seed dormancy prevents synchronized and uniform germination of a seed lot, which is clearly an unwanted trait in crops. Dormancy is the inability to germinate under optimal environmental conditions, allowing persistence in soil seed banks for extended periods of time, after which germination is completed only when environmental conditions are favourable for the establishment of a new plant generation [52]. Loss of seed dormancy was among the most common events towards domestication of legume crops [2]. Abscisic acid (ABA) is a positive regulator of seed dormancy, and is synthesised from carotenoid precursors [53]. In Arabidopsis, overexpression of a phytoene synthase gene led to the accumulation of carotenoids and ABA and to delayed germination [54]. Therefore, the decrease in carotenoids upon domestication could be related to the outbreeding of seed dormancy through down-regulation of the ABA synthesis pathway. In summary, carotenoids contents could be lower in crops because of alteration by humans of seed dispersal and seed dormancy.

Additionally, it should be considered that domestication implied the translocation of plants from natural environments to agro-ecosystems, where resources are generally plentiful and plant life is better buffered against environmental risks such as drought, nutrient deficiency or pathogens. In that sense, carotenoids on seeds may have decreased because, e.g., less oxidative stress because of improved, and more predictable, supply of water and nutrients might have relaxed selective pressures over their metabolic pathways.

### Lipophilic antioxidants, fatty acids and seed viability

In photosynthetic tissues the ratio between tocopherols and carotenoids apparently evolved towards higher tocopherols contents [55]. Apart from other roles, both tocopherols and carotenoids are lipophilic antioxidants. Thus, tocopherols and carotenoids complement one another and act synergistically. In some cases it has been shown that plants trend to compensate for the absence of one by the overproduction of the other. For example, in Arabidopsis the absence of zeaxanthin could be compensated for by higher levels of tocopherol [56] and *vice versa* [57]. Therefore, it is reasonable to assume that the lower carotenoids levels found in crops (Figure 1A) were compensated for by higher tocopherols levels. However, we found no evidence that the changes in tocopherols upon domestication were related to the changes in carotenoids (Figure 1C).

The composition and degree of saturation of membrane FAs determine their susceptibility to oxidation. In seeds, carotenoids and tocopherols are the main lipid soluble antioxidants that protect membranes and oil bodies from oxidation. In recalcitrant (i.e. desiccation sensitive) seeds high levels of C18:3 and low levels of tocopherol were correlated with oxidative damage and lower storability [22]. In orthodox (i.e. desiccation-tolerant) seeds, such as those studied here, diunsaturated FAs were suggested to facilitate survival in the desiccated state by improving membrane properties, increasing their elasticity and facilitating folding without loss of integrity [58,59]. In most of the domesticated species analysed we observed an increase in total SFAs, MUFAs and diunsaturated FAs (linoleic acid) whereas linolenic acid decreased (Figure 3B, Additional file 4). Overall, these changes in FAs composition could have made membranes less prone to oxidation (due to decreased linolenic acid), thereby relaxing the selection pressure for higher tocopherols levels to compensate for the lower carotenoid contents, whereby the increased MUFAs and linoleic acid could have contributed to the maintenance of membrane fluidity.

### Lipophilic antioxidants and fatty acids as nutrients

Fruits, vegetables and seeds are the primary dietary sources of carotenoids (including the provitamin A) and tocopherols (vitamin E), which play pivotal roles in the prevention of inflammatory processes, and coronary, neuromuscular and visual disorders. Nevertheless, vitamin A levels in the seeds of the most abundantly consumed staple crops are insufficient to meet minimum nutritional requirements [60] if not compensated for by dietary carotenoids intake from fruits and vegetables. Efforts are being made to increase carotenoids synthesis in seed crops by genetic engineering [61-63]. Alternatively, hybridization of crops with wild progenitors could represent a complementary tool for enhancing the carotenoids contents in edible seeds.

Most animals and humans require dietary intake of plant lipids as major source of calories and essential FAs [64]. A high ratio of UFAs to SFAs in the diet helps preventing cardiovascular diseases [37]. Unexpectedly, we found that the ratio of UFAs to SFAs decreased upon domestication. However, FA composition also affects seed taste. The by-products of lipid peroxidation, resulting in rancidification, have unpleasant odours or flavours [65,66]. Hence, the decrease in PUFAs, which are most susceptible to oxidation, that accompanied domestication of grain legumes (Figure 3) may have resulted from directed human selection for better flavour after storage.

## Conclusions

In summary, domestication of grain legumes was accompanied by a reduction in carotenoids contents, including molecules that are essential to human nutrition. Additionally, for many of the analysed species, domestication was also accompanied by a decrease in linolenic acid content. These changes are likely side-effects of the selection for other desired traits such as the loss of seed dispersal mechanisms and dormancy, and selection for seed storability and taste. In turn, the wild progenitors may still offer untapped potential for future improvement of the nutritional value of edible seeds. This novel work may serve as baseline for future studies in other taxa and plant organs to broaden our knowledge on the consequences of domestication in the nutritious value and fitness of crop-plants.

### Availability of supporting data

Supporting data include details on seed suppliers (Additional file 1), composition of individual carotenoids and tocopherols splitted by genera (Additional file 2) and their percentage of change following domestication (Additional file 4), and ratios of fatty acids to seed dry mass and total oil content (Additional file 3). All the supporting data are included as additional files.

## Additional files

Additional file 1:
**Detailed information on all seed lots studied.** Common and scientific names, tribes (all tribes were in the subfamily Papilionoideae), domestication status (D: domesticated; W: wild), accession identifier, seed origin information, and literature regarding wild progenitor assignment (superscript numbers) are provided. Seed suppliers (TAMU: Texas A&M University, USA; PRI: Peanut Research Institute, Shangdong, China; ICARDA: International Center for Agricultural Research in Dry Areas-FAO, Syria; CRF: Centro Nacional de Recursos Fitogenéticos-INIA, Spain; IPK: Germplasm bank of the Leibniz Institute of Plant Genetics and Crop Plant Research, Germany; WSU: Washington State University, USA; La Orden: Centro de Investigación Agraria Finca La Orden, Junta de Extremadura, Spain; UC: University of California, USA; JIC: John Innes Centre of excellence in plant science and microbiology, United Kingdom; Fitó: Semillas Fito international company, Spain; IITA: International Institute of Tropical Agriculture, Nigeria; CCIA: California Crop Improvement Association, USA. Accession identifiers refer to the code assigned by each seed supplier (NA: non aplicable). Country of origin refers to the country where the seeds were collected. Additional file 2:
**Carotenoids and tocopherols composition (μg g** ^**−1**^ **DM) in the seeds of grain legumes and their closest wild relatives (D, domesticated; W, wild).** Data are promedia (upper part) and standard error (lower part of the table) of 5 independent replicates. Additional file 3:
**Variation in the contents of individual seed carotenoids and tocopherols following domestication.** Variations (Δ%) are expressed as percentages of the contents in the wild relatives. Each data point represents one genus. The number (n) of genera for which the calculation of D% was possible is shown at the bottom of the panel. Additional file 4:
**Total oil content (% of seed DM) and fatty acid composition (expressed both as percentage of seed DM and of percentage of total oil content) in the seeds of grain legumes and their closest wild relatives (D, domesticated; W, wild).** Asterisks denote significant differences between grain legumes and their wild relatives (Mann–Whitney-U test, P < 0.05). Data are means ± SE (n ≥ 4). Significant decreases following domestication are highlighted in red and increases in green.

## Abbreviations

ABA: Abscisic acid
D: Domesticated
DM: Dry mass
FA: Fatty acid
FAMEs: Fatty acid methyl esters
MUFA: Monounsaturated fatty acid
PUFA: Poly unsaturated fatty acid
ROS: Reactive oxygen species
SFA: Saturated fatty acid
UFA: Unsaturated fatty acid
W: Wild
