# Supplementary material for: Side-effects of domestication: cultivated legume seeds contain similar tocopherols and fatty acids but less carotenoids than their wild counterparts
Source: BMC Plant Biol. 2014 Dec 20;14:1599. doi: 10.1186/s12870-014-0385-1 (PMC4302433; doi:10.1186/s12870-014-0385-1)
Supplement: Additional file 1: — Detailed information on all seed lots studied. Common and scientific names, tribes (all tribes were in the subfamily Papilionoideae), domestication status (D: domesticated; W: wild), accession identifier, seed origin information, and literature regarding wild progenitor assignment (superscript numbers) are provided. Seed suppliers (TAMU: Texas A&M University, USA; PRI: Peanut Research Institute, Shangdong, China; ICARDA: International Center for Agricultural Research in Dry Areas-FAO, Syria; CRF: Centro Nacional de Recursos Fitogenéticos-INIA, Spain; IPK: Germplasm bank of the Leibniz Institute of Plant Genetics and Crop Plant Research, Germany; WSU: Washington State University, USA; La Orden: Centro de Investigación Agraria Finca La Orden, Junta de Extremadura, Spain; UC: University of California, USA; JIC: John Innes Centre of excellence in plant science and microbiology, United Kingdom; Fitó: Semillas Fito international company, Spain; IITA: International Institute of Tropical Agriculture, Nigeria; CCIA: California Crop Improvement Association, USA. Accession identifiers refer to the code assigned by each seed supplier (NA: non aplicable). Country of origin refers to the country where the seeds were collected. [file 12870_2014_385_MOESM1_ESM.doc]

**Additional file 1.** Detailed information on all seed lots studied. Common and scientific names, tribes (all tribes were in the subfamily Papilionoideae), domestication status (D: domesticated; W: wild), accession identifier, seed origin information, and literature regarding wild progenitor assignment (superscript numbers) are provided. Seed suppliers (**TAMU**: Texas A&M University, USA; **PRI**: Peanut Research Institute, Shangdong, China; **ICARDA**: International Center for Agricultural Research in Dry Areas-FAO, Syria; **CRF**: Centro Nacional de Recursos Fitogenéticos-INIA, Spain; **IPK**: Germplasm bank of the Leibniz Institute of Plant Genetics and Crop Plant Research, Germany; **WSU:** Washington State University, USA; **La Orden**: Centro de Investigación Agraria Finca La Orden, Junta de Extremadura, Spain; **UC**: University of California, USA; **JIC**: John Innes Centre of excellence in plant science and microbiology, United Kingdom; **Fitó**: Semillas Fito international company, Spain; **IITA**: International Institute of Tropical Agriculture, Nigeria; **CCIA**: California Crop Improvement Association, USA. Accession identifiers refer to the code assigned by each seed supplier (NA: non aplicable). Country of origin refers to the country where the seeds were collected.

Literature regarding wild progenitor assignment

**^1^** **Seijo GJ, Lavia GI, Fernandez A, Krapovickas A, Ducasse E, Bertioli DJ, Moscone DEA**. 2007. Genomic relationships between the cultivated peanut (*Arachis hypogaea* – Leguminosae) and its close relatives revealed by double GISH. *American Journal of Botany* **94**, 1963-1971.

**^2^** **Sauer JD**. 1993. *Historical geography of crop plants. A select roster.* CRC Press. Boca Raton, USA.

**^3^** **Hymowitz T, Newell CA.** 1981. Taxonomy of the genus *Glycine*, domestication and uses of soybeans. *Economic Botany* **35**, 272-288.

**^4^** **Sarker A, Abd El Moneim A, Maxted N.** 2001. Grasspea and chicklinks (*Lathyrus* L.). In: Maxted N, Bennett J, eds. *Plant Genetic Resources of Legumes in the Mediterranean*. Kluwer Academic Publishers, 159-180.

**^5^** **Hancock JF** .2004. *Plant Evolution and the origin of crop species*. CABI Publishing.

**^6^** **Wolko B, Clements JC, Naganowska B, Nelson MN, Yang H**. 2011. *Lupinus*. In: Kole C, ed. *Wild crop relatives: genomic and breeding resources, legume crops and forages.* Springer 153-206.

**^7^** **Tomooka N, Kaga A, Isemura T, Vaughan D.** 2011. *Vigna*. In: Kole C, ed. *Wild crop relatives: genomic and breeding resources, legume crops and forages.* Springer 291-311.

| **Common name** | **Tribe** | **Scientific name** | **Domestication status** | **Accession identifier** | **Seed suppliers** | **Country of origin** |
| --- | --- | --- | --- | --- | --- | --- |
| Peanut^1^ | Dalbergieae | Arachis monticola | W | PI497261 | TAMU | Argentina |
|  |  | *Arachis hypogaea* | D | PI540478 | PRI | China |
| Chickpea^2^ | Cicerae | *Cicer reticulatum* | W | IG72945 ILWC116 | ICARDA | Turkey |
|  |  | Cicer arietinum | D | BGE024684 | CRF | Spain |
| Soybean^3^ | Phaseoleae | *Glycine soja* | W | 1039 | IPK | Russia |
|  |  | Glycine max | D | BGE010200 | CRF | Japan |
| Vetch^4^ | Fabeae | *Lathyrus cicera* | W | BGE022222 | CRF | Spain |
|  |  | *Lathyrus sativus* | D | IG 65060 IFLA 341 | ICARDA | Greece |
| Lentil^5^ | Fabeae | *Lens culinaris* subsp*. orientalis* | W | PI 572391 | WSU | Cyprus |
|  |  | *Lens culinaris* | D | BGE024690 | CRF | Spain |
| Lupin^6^ | Genisteae | *Lupinus luteus* | W | LO4570 | La Orden | Spain |
|  |  | *Lupinus luteus* | D | LO4500 | La Orden | Spain |
| Bean^2^ | Phaseoleae | *Phaseolus lunatus* | W | PI260406 | UC | Peru |
|  |  | *Phaseolus lunatus* | D | PI347798 | UC | Colombia |
| Pea^5^ | Fabeae | *Pisum sativum* | W | 267 | JIC | Greece |
|  |  | *Pisum sativum* | D | NA | Fitó | Spain |
| Faba bean^5^ | Fabeae | *Vicia narbonensis* | W | BGE013234 | CRF | Spain |
|  |  | *Vicia faba* | D | BGE011505 | CRF | Spain |
| Cowpea^7^ | Phaseoleae | *Vigna unguiculata* subsp*. unguiculata* | W | PI447516 | IITA | Nigeria |
|  |  | *Vigna unguiculata* | D | PI599213 | CCIA | USA |
