# Supplementary material for: Side-effects of domestication: cultivated legume seeds contain similar tocopherols and fatty acids but less carotenoids than their wild counterparts
Source: BMC Plant Biol. 2014 Dec 20;14:1599. doi: 10.1186/s12870-014-0385-1 (PMC4302433; doi:10.1186/s12870-014-0385-1)
Supplement: Additional file 2: — Carotenoids and tocopherols composition (μg g −1 DM) in the seeds of grain legumes and their closest wild relatives (D, domesticated; W, wild). Data are promedia (upper part) and standard error (lower part of the table) of 5 independent replicates. [file 12870_2014_385_MOESM2_ESM.doc]

**Additional file 2** Carotenoids and tocopherols composition (μg g^-1^DM) in the seeds of grain legumes and their closest wild relatives (D, domesticated; W, wild). Data are promedia (upper part) and standard error (lower part of the table) of 5 independent replicates.

|  |  |  | **Carotenoids** | | | | | | | | **Tocopherols** | | | |
| --- | --- | --- | --- | --- | --- | --- | --- | --- | --- | --- | --- | --- | --- | --- |
|  | **Domestication** | ***Genera*** | **Neoxanthin** | **Violaxanthin** | **Lutein epoxide** | **Antheraxanthin** | **Lutein** | **Zeaxanthin** | **α-Carotene** | **β-Carotene** | **α-T** | **β-T** | **γ-T** | **δ-T** |
| Promedia | D | *Arachis* | 0 | 0 | 0 | 0 | 0.18 | 0.00 | 0 | 0.14 | 4.30 | 0.10 | 14.99 | 4.10 |
|  | D | *Cicer* | 0 | 0 | 0 | 0 | 3.77 | 5.49 | 0 | 0.19 | 18.91 | 0 | 45.96 | 0 |
|  | D | *Glycine* | 0 | 0 | 0 | 0 | 5.61 | 0.73 | 0 | 0.11 | 11.68 | 0.75 | 74.58 | 59.87 |
|  | D | *Lathyrus* | 0 | 0 | 0 | 0 | 6.19 | 0.97 | 0 | 0 | 0 | 0 | 8.74 | 0 |
|  | D | *Lens* | 0 | 0 | 0 | 0 | 7.26 | 2.82 | 0 | 0 | 0 | 0 | 9.13 | 0 |
|  | D | *Lupinus* | 0 | 0 | 0 | 0 | 3.02 | 0.99 | 0 | 0.12 | 0 | 0 | 14.45 | 0 |
|  | D | *Phaseolus* | 0 | 0 | 0 | 0 | 0.08 | 0.00 | 0 | 0 | 0 | 0 | 8.25 | 0 |
|  | D | *Psium* | 0 | 0 | 0 | 0 | 2.76 | 0.48 | 0 | 0.44 | 0 | 0 | 29.01 | 2.79 |
|  | D | *Vicia* | 0 | 0 | 0 | 0 | 3.01 | 0.39 | 0 | 0 | 0 | 0 | 9.37 | 0 |
|  | D | *Vigna* | 0 | 0 | 0 | 0 | 0.61 | 0.00 | 0 | 0 | 0 | 0 | 10.78 | 48.70 |
|  | W | *Arachis* | 0 | 0 | 0 | 0 | 0.89 | 0.00 | 0 | 0 | 88.28 | 0 | 31.97 | 0 |
|  | W | *Cicer* | 0 | 0 | 0 | 0.72 | 7.62 | 8.33 | 0 | 0.91 | 4.75 | 0 | 27.47 | 1.87 |
|  | W | *Glycine* | 2.02 | 1.02 | 0.59 | 0.76 | 17.48 | 1.71 | 0 | 3.75 | 7.93 | 0.14 | 35.18 | 27.31 |
|  | W | *Lathyrus* | 0 | 0.23 | 0.13 | 0.32 | 7.64 | 0.98 | 0 | 0 | 0 | 0 | 9.06 | 0 |
|  | W | *Lens* | 0 | 0 | 0 | 0.49 | 10.74 | 5.45 | 0 | 0.97 | 3.88 | 0 | 19.37 | 0 |
|  | W | *Lupinus* | 0.11 | 0 | 0 | 0 | 4.32 | 1.37 | 0 | 1.67 | 0 | 0 | 22.70 | 0 |
|  | W | *Phaseolus* | 0 | 0 | 0 | 0 | 0.17 | 0.00 | 0 | 0 | 0 | 0 | 13.77 | 0 |
|  | W | *Psium* | 0 | 0 | 0 | 0 | 7.77 | 0.71 | 0 | 0.07 | 0 | 0 | 24.66 | 6.80 |
|  | W | *Vicia* | 0 | 0 | 0 | 0 | 3.23 | 0.23 | 0 | 0 | 0 | 0 | 6.29 | 0.14 |
|  | W | *Vigna* | 0 | 0.04 | 0.41 | 1.08 | 0.29 | 0.43 | 0 | 0 | 0 | 0 | 9.43 | 55.77 |
| SE | D | *Arachis* | 0 | 0 | 0 | 0 | 0.09 | 0 | 0 | 0.07 | 1.79 | 0.10 | 2.40 | 0.69 |
|  | D | *Cicer* | 0 | 0 | 0 | 0 | 0.97 | 1.44 | 0 | 0.09 | 1.91 | 0 | 6.99 | 0 |
|  | D | *Glycine* | 0 | 0 | 0 | 0 | 0.96 | 0.21 | 0 | 0.11 | 0.84 | 0.24 | 2.52 | 5.13 |
|  | D | *Lathyrus* | 0 | 0 | 0 | 0 | 0.78 | 0.12 | 0 | 0 | 0 | 0 | 0.94 | 0 |
|  | D | *Lens* | 0 | 0 | 0 | 0 | 0.82 | 0.36 | 0 | 0 | 0 | 0 | 0.62 | 0 |
|  | D | *Lupinus* | 0 | 0 | 0 | 0 | 0.24 | 0.07 | 0 | 0.04 | 0 | 0 | 0.95 | 0 |
|  | D | *Phaseolus* | 0 | 0 | 0 | 0 | 0.08 | 0 | 0 | 0 | 0 | 0 | 0.93 | 0 |
|  | D | *Psium* | 0 | 0 | 0 | 0 | 0.50 | 0.15 | 0 | 0.09 | 0 | 0 | 2.13 | 0.17 |
|  | D | *Vicia* | 0 | 0 | 0 | 0 | 0.47 | 0.07 | 0 | 0 | 0 | 0 | 0.22 | 0 |
|  | D | *Vigna* | 0 | 0 | 0 | 0 | 0.05 | 0 | 0 | 0 | 0 | 0 | 0.78 | 2.00 |
|  | W | *Arachis* | 0 | 0 | 0 | 0 | 0.20 | 0 | 0 | 0 | 25.36 | 0 | 3.38 | 0 |
|  | W | *Cicer* | 0 | 0 | 0 | 0.15 | 1.01 | 1.07 | 0 | 0.25 | 0.84 | 0 | 2.87 | 0.78 |
|  | W | *Glycine* | 0.19 | 0.07 | 0.03 | 0.09 | 0.92 | 0.08 | 0 | 0.34 | 0.57 | 0.14 | 1.14 | 0.75 |
|  | W | *Lathyrus* | 0 | 0.06 | 0.04 | 0.08 | 0.74 | 0.08 | 0 | 0 | 0 | 0 | 0.50 | 0 |
|  | W | *Lens* | 0 | 0 | 0 | 0.16 | 1.10 | 0.82 | 0 | 0.36 | 0.59 | 0 | 1.87 | 0 |
|  | W | *Lupinus* | 0.11 | 0 | 0 | 0 | 0.60 | 0.24 | 0 | 0.28 | 0 | 0 | 1.29 | 0 |
|  | W | *Phaseolus* | 0 | 0 | 0 | 0 | 0.02 | 0 | 0 | 0 | 0 | 0 | 0.97 | 0 |
|  | W | *Psium* | 0 | 0 | 0 | 0 | 1.58 | 0.18 | 0 | 0.05 | 0 | 0 | 0.84 | 1.72 |
|  | W | *Vicia* | 0 | 0 | 0 | 0 | 0.24 | 0.02 | 0 | 0 | 0 | 0 | 0.56 | 0.14 |
|  | W | *Vigna* | 0 | 0.04 | 0.05 | 0.29 | 0.17 | 0.04 | 0 | 0 | 0 | 0 | 0.80 | 3.77 |
