# Supplementary material for: Side-effects of domestication: cultivated legume seeds contain similar tocopherols and fatty acids but less carotenoids than their wild counterparts
Source: BMC Plant Biol. 2014 Dec 20;14:1599. doi: 10.1186/s12870-014-0385-1 (PMC4302433; doi:10.1186/s12870-014-0385-1)

## Additional file 3 Variation in the contents of individual seed carotenoids and tocopherols following domestication. Variations (Δ%) are expressed as percentages of the contents in the wild relatives. Each data point represents one genus. The number (n) of genera for which the calculation of Δ% was possible is shown at the bottom of the panel.


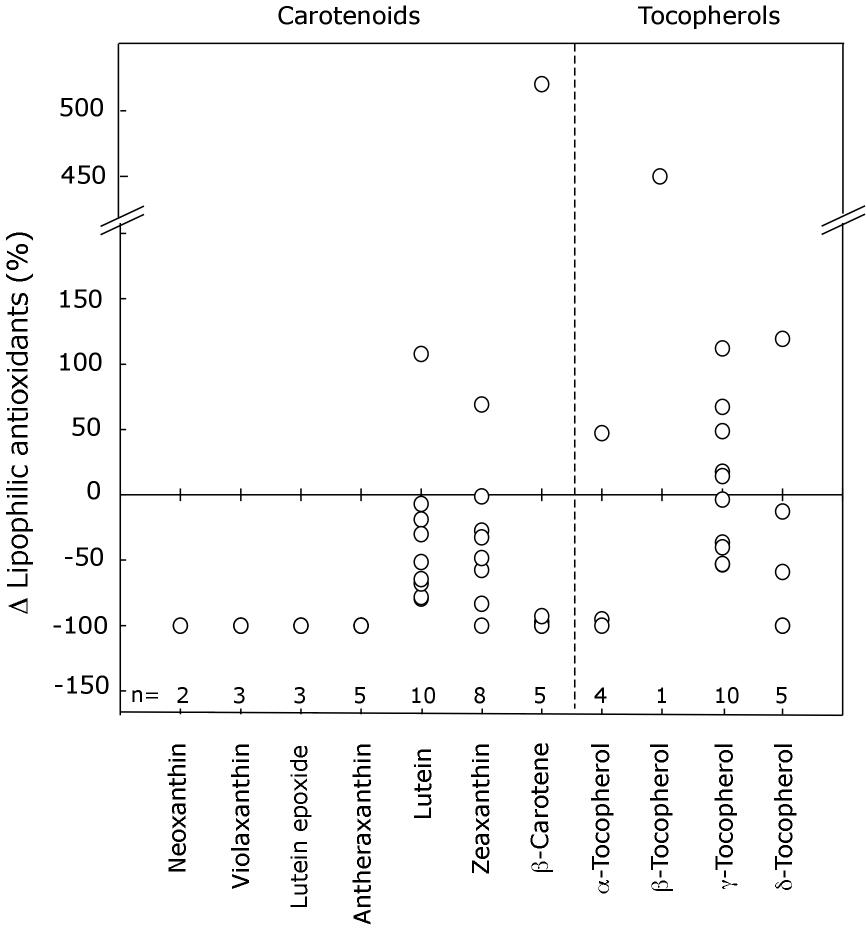

Supplement: Additional file 3: — Variation in the contents of individual seed carotenoids and tocopherols following domestication. Variations (Δ%) are expressed as percentages of the contents in the wild relatives. Each data point represents one genus. The number (n) of genera for which the calculation of D% was possible is shown at the bottom of the panel. [file 12870_2014_385_MOESM3_ESM.doc]
