# Supplementary material for: Side-effects of domestication: cultivated legume seeds contain similar tocopherols and fatty acids but less carotenoids than their wild counterparts
Source: BMC Plant Biol. 2014 Dec 20;14:1599. doi: 10.1186/s12870-014-0385-1 (PMC4302433; doi:10.1186/s12870-014-0385-1)
Supplement: Additional file 4: — Total oil content (% of seed DM) and fatty acid composition (expressed both as percentage of seed DM and of percentage of total oil content) in the seeds of grain legumes and their closest wild relatives (D, domesticated; W, wild). Asterisks denote significant differences between grain legumes and their wild relatives (Mann–Whitney-U test, P < 0.05). Data are means ± SE (n ≥ 4). Significant decreases following domestication are highlighted in red and increases in green. [file 12870_2014_385_MOESM4_ESM.doc]

**Additional file 4.** Total oil content (% of seed DM) and fatty acid composition (expressed both as percentage of seed DM and of percentage of total oil content) in the seeds of grain legumes and their closest wild relatives (D, domesticated; W, wild). Asterisks denote significant differences between grain legumes and their wild relatives (Mann-Whitney-U test, *P*<0.05). Data are means ± SE (n≥4). Significant decreases following domestication are highlighted in red and increases in green.

|  |  | **(% of seed DM)** | | | | **(% of total oil content)** | | |
| --- | --- | --- | --- | --- | --- | --- | --- | --- |
| **Species** | **Domestication status** | **Oil content** | **SFAs** | **MUFAs** | **PUFAs** | **SFAs** | **MUFAs** | **PUFAs** |
| Arachis hypogea | W | 61.19 ± 2.89 | 12.78 ± 0.52 | 22.69 ± 1.45 | 25.59 ± 1.07 | 20.95 ± 0.5 | 36.92 ± 0.7 | 41.91 ± 0.7 |
| *Arachis monticola* | D | 59.44 ± 2.17 | 11.11 ± 0.33* | 28.00 ± 0.99* | 18.93 ± 0.90* | 18.72 ± 0.2* | 47.14 ± 0.7* | 31.78 ± 0.5* |
| *Cicer arietinum* | W | 4.75 ± 0.14 | 0.85 ± 0.02 | 1.16 ± 0.01 | 2.71 ± 0.14 | 17.91 ± 0.4 | 24.61 ± 0.9 | 56.94 ± 1.1 |
| *Cicer reticulatum* | D | 5.90 ± 0.11* | 0.94 ± 0.01* | 1.49 ± 0.03* | 3.43 ± 0.07* | 15.99 ± 0.2* | 25.16 ± 0.2 | 58.16 ± 0.2 |
| *Glycine max* | W | 12.22 ± 0.40 | 2.09 ± 0.06 | 1.29 ± 0.05 | 8.83 ± 0.31 | 17.13 ± 0.1 | 10.61 ± 0.3 | 72.26 ± 0.4 |
| *Glycine soja* | D | 23.06 ± 0.46* | 3.90 ± 0.09* | 5.95 ± 0.14* | 13.21 ± 0.40* | 16.90 ± 0.3 | 25.84 ± 0.7* | 57.25 ± 0.9* |
| *Lathyrus sativus* | W | 1.37 ± 0.07 | 0.30 ± 0.01 | 0.15 ± 0.02 | 0.92 ± 0.05 | 22.16 ± 0.7 | 10.85 ± 0.9 | 66.98 ± 0.6 |
| *Lathyrus cicera* | D | 1.65 ± 0.06* | 0.32 ± 0.01 | 0.23 ± 0.03 | 1.11 ± 0.03* | 19.19 ± 0.5* | 13.47 ± 1.4 | 67.35 ± 1.0 |
| *Lens culinaris* | W | 2.50 ± 0.08 | 0.51 ± 0.02 | 0.53 ± 0.02 | 1.46 ± 0.05 | 20.46 ± 0.2 | 21.32 ± 0.3 | 58.23 ± 0.2 |
| *Lens culinaris* | D | 2.79 ± 0.13 | 0.58 ± 0.02 | 0.76 ± 0.04* | 1.45 ± 0.07 | 20.74 ± 0.2 | 27.11 ± 0.3* | 52.15 ± 0.2* |
| *Lupinus luteus* | W | 5.73 ± 0.13 | 0.93 ± 0.03 | 1.63 ± 0.06 | 3.17 ± 0.09 | 16.19 ± 0.6 | 28.44 ± 1.1 | 55.36 ± 0.6 |
| *Lupinus luteus* | D | 5.04 ± 0.19* | 0.97 ± 0.04 | 1.13 ± 0.05* | 2.94 ± 0.11 | 19.22 ± 0.2* | 22.41 ± 0.6* | 58.37 ± 0.7* |
| *Phaseolus lunatus* | W | 1.47 ± 0.06 | 0.32 ± 0.01 | 0.06 ± 0.01 | 1.09 ± 0.05 | 21.91 ± 0.3 | 3.77 ± 0.3 | 74.31 ± 0.3 |
| *Phaseolus lunatus* | D | 2.40 ± 0.12* | 0.79 ± 0.04* | 0.24 ± 0.03* | 1.37 ± 0.05* | 32.87 ± 0.4* | 9.72 ± 1.1* | 57.41 ± 1.0* |
| *Pisum sativum* | W | 2.30 ± 0.08 | 0.39 ± 0.01 | 0.38 ± 0.05 | 1.54 ± 0.03 | 16.81 ± 0.5 | 16.28 ± 1.7 | 66.91 ± 1.2 |
| *Pisum sativum* | D | 3.78 ± 0.06* | 0.68 ± 0.03* | 0.79 ± 0.03* | 2.31 ± 0.04* | 17.92 ± 0.6 | 20.97 ± 0.9* | 61.11 ± 1.0* |
| *Vicia faba* | W | 2.06 ± 0.07 | 0.31 ± 0.01 | 0.48 ± 0.03 | 1.27 ± 0.05 | 15.20 ± 0.3 | 23.42 ± 1.4 | 61.39 ± 1.2 |
| *Vicia narbonensis* | D | 1.90 ± 0.04 | 0.37 ± 0.01* | 0.32 ± 0.01* | 1.21 ± 0.04 | 19.60 ± 0.3* | 16.72 ± 0.7* | 63.68 ± 0.7 |
| *Vigna unguiculata* | W | 2.69 ± 0.02 | 0.78 ± 0.02 | 0.09 ± 0.01 | 1.82 ± 0.02 | 29.11 ± 0.5 | 3.18 ± 0.2 | 67.71 ± 0.7 |
| *Vigna unguiculata* | D | 2.55 ± 0.05 | 0.84 ± 0.02* | 0.19 ± 0.01* | 1.52 ± 0.03* | 32.99 ± 0.2* | 7.46 ± 0.3* | 59.54 ± 0.4* |

## 
